# 

**Published:** 1885-12

**Authors:** 


ORIGINAL COMMUNICATIONS:

| | PAGE. |
|---|---|
| Toxic Properties of Sassafras. J. Bartlett | 499 |
| Spiritualism and Hypnotism. G. C. Paoli | 505 |
| Intubation of the Larynx. F. E. Waxham | 511 |
| Two Hundred Cases of Tonsilitis. J. M. G. Carter | 514 |
| Glaucoma Treated with Eserine. S. O. Richey | 519 |
| Blindness following Hæmorrhage. B. Bettman | 525 |

EDITORIAL:

| | |
|---|---|
| The Ninth International Medical Congress | 532 |
| The American Academy of Medicine | 556 |
| The Annual Report for 1885 of the Surgeon-General of the Army | 557 |
| The Annual Report for 1885 of the Surgeon-General of the Navy | 558 |

DOMESTIC CORRESPONDENCE:

| | |
|---|---|
| Unusual Distension of the Urinary Bladder. F. A. Butterfield | 560 |
| Fœtus Compressus. M. F. Porter | 561 |

SOCIETY REPORTS.

| | PAGE. |
|---|---|
| *Chicago Medical Society, November 4th.* Nervous Paroxysm or Syncope. H. T. Byford | 562 |
| Palatable Therapeutics. F. H. Martin | 565 |
| *Chicago Medical Society, November 16th.* Two Hundred Cases of Tonsilitis. J. M. G. Carter | 571 |
| *The American Academy of Medicine* | 577 |
| *The Philadelphia Academy of Surgery.* Foreign Body Causing Vesical Calculus. J. E. Mears | 580 |
| Excision of the Scapula. J. Brinton | 582 |
| Congenital Malformation of the Colon. C. B. Nanerede | 583 |
| Treatment of Carbuncle | 584 |
| Nitrous Oxide Gas in Examination of Fractures. J. M. Barton | 585 |

BOOK REVIEWS:

| | |
|---|---|
| Fownes' Manual of Chemistry | 587 |
| A Complete Pronouncing Medical Dictionary. J. Thomas | 587 |
| Text-Book of Pharmacology, Therapeutics and Materia Medica. T. L. Brunton | 588 |

ITEMS:

| | |
|---|---|
| A New Journal | 588 |

